# Supplementary material for: Suicidal Ideation in the Australian Construction Industry: Prevalence and the Associations of Psychosocial Job Adversity and Adherence to Traditional Masculine Norms
Source: Int J Environ Res Public Health. 2022 Nov 26;19(23):15760. doi: 10.3390/ijerph192315760 (PMC9738943; doi:10.3390/ijerph192315760)
Supplement: Supplementary file 1 [file ijerph-19-15760-s001.zip › ijerph-2039286-supplementary.pdf]

Supplementary Materials

1) **Supplementary File S1.** Psychosocial Job Adversity QuestionsMy job is more stressful than I had ever imagined
2) I fear the amount of stress in my job will make me physically ill
3) I get paid fairly for the things I do in my job
4) I have a secure future in my job
5) The company I work for will still be in business 5 years from now
6) I worry about the future of my job
7) My job is complex and difficult
8) My job often requires me to learn new skills
9) I use many of my skills and abilities in my current job
10) I have a lot of freedom to decide how I do my own work
11) I have a lot to say about what happens on my job
12) I have a lot of freedom to decide when I do my work

Supplementary File S2. Conforming to Masculine Norms Inventory 22 Questions

1) My work/school is the most important part of my life
2) I make sure people do as I say
3) In general, I do not like risky situations
4) It would be awful if someone thought I was gay
5) I love it when men are in charge of women
6) I like to talk about my feelings
7) I would feel good if I had many sexual partners
8) It is important to me that people think I am heterosexual
9) I believe that violence is never justified
10) I tend to share my feelings
11) I should be in charge
12) I would hate to be important
13) Sometimes violent action is necessary
14) I don't like giving all my attention to work/school
15) More often than not, losing does not bother me
16) If I could, I would frequently change sexual partners
17) I never do things to be an important person
18) I never ask for help
19) I enjoy taking risks
20) Men and women should respect each other as equals
21) Winning is the most important thing
22) It bothers me when I have to ask for help
